# The association between urban land use and depressive symptoms in young adulthood: a FinnTwin12 cohort study

**DOI:** 10.1038/s41370-023-00619-w

**Published:** 2023-12-11

**Authors:** Zhiyang Wang, Alyce M. Whipp, Marja Heinonen-Guzejev, Maria Foraster, Jordi Júlvez, Jaakko Kaprio

**Affiliations:** 1 Institute for Molecular Medicine Finland, Helsinki Institute of Life Science, University of Helsinki, Helsinki, Finland; 2 Department of Public Health, University of Helsinki, Helsinki, Finland; 3 PHAGEX Research Group, Blanquerna School of Health Science, Universitat Ramon Llull (URL), Barcelona, Spain; 4 ISGlobal-Instituto de Salud Global de Barcelona Campus MAR, Parc de Recerca Biomèdica de Barcelona (PRBB), Barcelona, Spain; 5 Universitat Pompeu Fabra (UPF), Barcelona, Spain; 6 CIBER Epidemiología y Salud Pública (CIBEREsp), Madrid, Spain; 7 Clinical and Epidemiological Neuroscience (NeuroÈpia), Institut d’Investigació Sanitària Pere Virgili (IISPV), Reus, Spain

**Keywords:** Land use, Depressive symptoms

## Abstract

**Background:**

Depressive symptoms lead to a serious public health burden and are considerably affected by the environment. Land use, describing the urban living environment, influences mental health, but complex relationship assessment is rare.

**Objective:**

We aimed to examine the complicated association between urban land use and depressive symptoms among young adults with differential land use environments, by applying multiple models.

**Methods:**

We included 1804 individual twins from the FinnTwin12 cohort, living in urban areas in 2012. There were eight types of land use exposures in three buffer radii. The depressive symptoms were assessed through the General Behavior Inventory (GBI) in young adulthood (mean age: 24.1). First, K-means clustering was performed to distinguish participants with differential land use environments. Then, linear elastic net penalized regression and eXtreme Gradient Boosting (XGBoost) were used to reduce dimensions or prioritize for importance and examine the linear and nonlinear relationships.

**Results:**

Two clusters were identified: one is more typical of city centers and another of suburban areas. A heterogeneous pattern in results was detected from the linear elastic net penalized regression model among the overall sample and the two separated clusters. Agricultural residential land use in a 100 m buffer contributed to GBI most (coefficient: 0.097) in the “suburban” cluster among 11 selected exposures after adjustment with demographic covariates. In the “city center” cluster, none of the land use exposures was associated with GBI, even after further adjustment with social indicators. From the XGBoost models, we observed that ranks of the importance of land use exposures on GBI and their nonlinear relationships are also heterogeneous in the two clusters.

**Impact:**

- This study examined the complex relationship between urban land use and depressive symptoms among young adults in Finland. Based on the FinnTwin12 cohort, two distinct clusters of participants were identified with different urban land use environments at first. We then employed two pluralistic models, elastic net penalized regression and XGBoost, and revealed both linear and nonlinear relationships between urban land use and depressive symptoms, which also varied in the two clusters. The findings suggest that analyses, involving land use and the broader environmental profile, should consider aspects such as population heterogeneity and linearity for comprehensive assessment in the future.

## Introduction

Depressive symptoms are very common and reflect a chronic, complex, and multifactorial mental health condition. The burden of depressive symptoms is growing, especially among younger people. There has been a large rise in the incidence of depressive episodes or disorders among young adults across multiple countries [1–3]. The COVID-19 pandemic induced a negative mental health impact and increased the prevalence of depressive symptoms among young adults [4, 5]. Moreover, depressive symptoms were associated with a higher odds of risky behavior such as substance use and self-harm, which resulted in further psychological and physical health problems [6]. Although there is a genetic predisposition to occur more depressive symptoms, which a meta-analysis in 2020 estimated a heritability of 37% [7], several twin studies across countries have identified the vital role of environmental influences on mental health, including depressive symptoms among young adults, inspiring etiological consideration of various environments [8, 9].

Land use describes the human utilization of land, involving the transformation from undeveloped areas into residential and living environments. Urbanization is a pivotal driving force for the change of current land use systems [10], and urban planners consider multiple concepts such as suitability, competitiveness, need diversity, or resource scarcity to evaluate land use [11]. A recent UK biobank study identified specific urban environmental profiles including urban land use density that affect mental health through the regional brain volume and pertinent biological pathways [12]. A Finnish study found that variables referred to the urban environment including land use related to a low incidence of serious mental illnesses [13]. Therefore, advancing liveable initiatives and shaping diverse land use is able to promote healthy lifestyles, urban amenities, and nature conservation to ultimately improve human health [14, 15]. Some studies have specifically addressed the relationship between land use, via different indeces, and mental health/status, but their results were inconsistent [16–18]. Existing indices have some limitations, such as insensitiveness to capture the interaction between different types of land use [19]. Inconsistent evidence reflects the complexity of the land use effect, demanding further sophisticated analysis, while we will encounter difficulties such as high-dimensionality and small effect sizes [20]. Instead of conventional regression models with a single index, interpretable and robust multi-exposure models are recommended. Ohanyan and colleagues have applied some machine learning models, illustrated their characteristics, and employed them in a study on a wide range of urban exposures and type-2 diabetes [21, 22]. Some simulation and review studies have compared statistical approaches and assessed model performance [23–25]. However, this type of research is relatively rare on mental health.

To fulfill the current research gap, we hypothesized there is a complex relationship between land use, unable to be quantified by conventional indices, and depressive symptoms with three objectives: a) to cluster participants who shared a similar pattern of urban land use; b) to assess both the linear and nonlinear relationships between them in young adulthood; and c) to observe the possible differences in these relationships between clusters.

## Subjects and methods

### Study participants

The participants were from the FinnTwin12 cohort, which is a population-based prospective cohort among all Finnish twins born between 1983 and 1987, and their parents. At baseline, 5522 twins were invited and 5184 twins replied to our questionnaire (age 11–12, wave one), and they compose the overall cohort. All twins were invited to participate in the first follow-up survey with 92% retention at age 14 (wave two). Moreover, at age 14, 1035 families were invited to take part in an intensive substudy with psychiatric interviews, some biological samples, and additional questionnaires, and 1854 twins participated in these interviews. They were also invited to a second intensive survey as young adults, with a participation rate of 73% (*n* = 1347 individual twins), and completed the detailed young adulthood questionnaires and interviews (part of wave four). In addition, all of the twins in the overall cohort completed general age 17 questionnaires (wave three) and twins from the non-intensive study completed young adult questionnaires (wave four). Wave four was conducted from 2004 to 2012, in which overall 4824 individual twins were invited and 3404 replied. In this study, we included twins who participated in wave four. An updated review of this cohort was published recently [26].

### Measures

#### Depressive symptoms

In this study, the short-version General Behavior Inventory (GBI) was used to evaluate depressive symptoms among twins in young adulthood [27]. It is a self-reported inventory designed to identify mood-related behaviors, which is composed of 10 questions with a 4-point Likert scale from 0 (never) to 3 (very often) to query the occurrence of depressive symptoms [28]. The total score ranges from 0 to 30, and a higher score implies more depressive symptoms exist. To validate the GBI, we compared it to a Diagnostic and Statistical Manual of Mental Disorders-IV diagnosis of major depressive disorder (MDD) assessed by the Semi-Structured Assessment for the Genetics of Alcoholism (SSAGA) interview from the intensive study [29]. In a logistic regression model, the GBI score in young adulthood strongly predicted MDD, with the area under the receiver operating characteristic curve (AUC) of 0.8328 (among twins included in this study’s analysis).

#### Land use

The EUREF-FIN geocodes of twins from birth to 2021 were derived from the Digital and Population Data Services Agency, Finland. We used geocodes in 2012 to merge the land use exposures, derived from Urban Atlas (UA) 2012, to the twin data. UA is a part of land monitoring services to provide reliable, inter-comparable, high-resolution land use maps in the European Union and European Free Trade Association countries in 2006, 2012, and 2018 [30]. We used UA 2012 because it covers more areas, over 700 larger functional urban areas, and contains more detailed categories of land use information, compared to UA 2006. Land use exposures included the percentage of 8 types of land use (high-density residential, low-density residential, industrial and commercial, infrastructure, urban green, agricultural residential, natural, and water) in an area of 100, 300, and 500 m radius buffer zones for each geocode in urban Finland (total of 24 exposures).

Additionally, we also calculated the land use mix index in different buffers, which described the diversity of land uses through the Shannon’s Evenness Index. It provides information on area composition and richness, covering different land use types and their relative abundances. The equation is defined as follows [31]:

$${land\; use\; mix\; index}=\left(-\mathop{\sum }\limits_{i=1}^{n}{P}_{i}\times {{{{{\mathrm{ln}}}}}}\,{P}_{i}\right)/{{{{{\mathrm{ln}}}}}}\,n$$

*P*_*i*_ is the percentage of each type of land use in zone *i*; *n* is the number of land use types. It ranges from 0 to 1, and a higher value indicates a more balanced distribution of land between the different types of land use.

#### Covariates

Seven covariates (demographic) were defined a priori: sex (male, female), zygosity (monozygotic (MZ), dizygotic (DZ), unknown), parental education (limited, intermediate, high), smoking (never, former, occasional, current), work status (full-time, part-time, irregular, not working), secondary level school (vocational, senior high school, none), and age. The latter four variables came from the young adulthood survey. Parental education was based on maternal and paternal reports, while zygosity was based on DNA polymorphisms and/or a validated zygosity questionnaire [32].

Another four social indicators: age structure (proportion of people over age 18 in the total population), education level (bachelor´s/equivalent or above of the population over age 16 (%)), unemployment (unemployment rates among people who were between 25 and 54 years old (%)), and income level (proportion of households in highest income quartile in the country) were introduced to account for socioeconomic status segregation. We derived social indicators in 2012 at the postal code level of the twins’s residence at that time from Statistics Finland.

### Analysis

#### Preparation and description

We only included those twins who had available land use exposures in 2012 in urban areas (as defined above), indicating that they lived in the urban areas in Finland, and provided GBI assessment in young adulthood, in order to have a larger sample size and have the two measurements be as close as possible on the time scale. A total of 1804 individual twins (589 twin pairs and 626 individual twins) were included and the mean age in providing GBI assessment was 24.07 years (around 2007–2011). Due to the skewness of the GBI score, we added one to the GBI score and log-transformed it for the following analysis. A correlation matrix was drawn between land use exposures. Then, we proposed several approaches to assess the relationship between land use exposures and depressive symptoms.

#### Unsupervised clustering

To group twin individuals who have similar land use in an exploratory way, we used unsupervised K-means clustering. The K-means clustering method employs a non-hierarchical partitional algorithm. It calculats the total within-cluster variation as the sum of the squared Euclidean distance between each sample and the corresponding K-number random-assigned centroid in each cluster (*k*). *X*_*ik*_ is the *i*^th^ observation belonging to cluster (*k*= 1, 2, …., K) and *n*_*K*_ is the number of observations in cluster *k*. The overall within-cluster variation is defined as follows [33]:

$$\mathop{\sum }\limits_{k=1}^{K}\mathop{\sum }\limits_{i=1}^{{n}_{k}}{\left({X}_{{ik}}-\frac{1}{{n}_{K}}\mathop{\sum }\limits_{i=1}^{{n}_{k}}{X}_{{ik}}\right)}^{2}$$

The process stops when the criterion is met (smallest overall within-cluster variation) [33]. It is one of the simplest and fastest clustering methods, and is also able to handle outliers or inappropriate variables [34, 35]. Only the 24 land use exposures were included in the clustering algorithm. We used the Silhouette method to estimate the optimal number of pre-specified cluster [36], and two clusters were identified (Supplemental Fig. 1). The R package “Factoextra” was used [35].

#### Pluralistic analysis

We split the twin participants into training and testing subsets. In full twin pairs, we performed a 1:1 random split within the pair. The remaining individual twins all went into the training subset. The training sample size was 1215 and the testing sample size was 589, and the size in each cluster varied (Supplemental Table 1). By the splitting process, we do not need to consider the statistical effect of complex sampling cluster effects by twin pair status since all individuals in both samples are unrelated. We chose two types of models and adjusted covariates to evaluate the risk estimation of 24 land use exposures (*j*).

First, the linear elastic net penalized regression model was applied for feature selection, which uses a hybrid of the lasso and ridge penalized methods to fit the generalized linear model [37]. It encourages the grouping effect that correlated variables tend to be in or out of the model together with similar coefficients, and then variables are selected based on their predictive power in the context of penalty [38]. Coefficients are shrunk, even to zero, to promote sparsity and reduce multicollinearity [39]. It is very useful in datasets with highly correlated variables. A typical linear regression model based on N participants with the combined penalized term is defined as follows [39]:

$$\mathop{\min }\limits_{{\beta }_{0},\beta }\left(\frac{1}{2N}\mathop{\sum }\limits_{i=1}^{N}{\left({y}_{i}-{\beta }_{0}-{x}_{i}^{T}\beta \right)}^{2}+\lambda \mathop{\sum }\limits_{j=1}^{p}\left(\left(\frac{1-\alpha }{2}\right){\beta }_{j}^{2}+\alpha \left|{\beta }_{j}\right|\right)\right)$$

*y*_*i*_ is the dependent response and *x*_*i*_ is the independent factor at observation *i*. *λ* is a positive regularization parameter. *β*_0_ and *β* are scalar and p-vector coefficients, respectively. We set the *α*, ranging from 0.1 to 1.0, as a tuning parameter, for the penalty. The final models were selected by 10-fold cross-validation with minimal criteria to determine the optimal degree of penalization [37]. There were two adjustment plans: 1) demographic covariates (minimal), and 2) demographic covariates and social indicators (further). We forced the demographic covariates and social indicators into the models, without penalty, to fully adjust them. Stata package “elasticnet” was used.

Further, to assess the nonlinear relationship, the supervised machine learning model eXtreme Gradient Boosting (XGBoost) was used. It is a tree-based gradient boosting technique, utilizing the weights of trees, which is good at predicting and reduces the risk of overfitting [40, 41]. The objective function of XGBoost starts with two parts: a loss function and a regularization term, and we aim to obtain the optimal output value (*O*_*value*_) to minimize the function, defined as follows:

$$\mathop{\sum }\limits_{i=1}^{n}L\left({y}_{i},{p}_{i}^{t-1}+{O}_{{value}}\right)+\gamma T+\frac{1}{2}\lambda {O}_{{value}}^{2}$$

${p}_{i}^{t-1}$ is the previous prediciton of tree *t* at observation *i*. *T* is the number of leaf nodes in a tree, and *γ* and *λ* are the definable penalty factors to avoid overfitting. Then, we rewrite the loss function according to the 2nd Taylor Approximation:

$$L\left({y}_{i},{P}_{i}^{t-1}+{O}_{{value}}\right)\approx \, 	 L\left(y,{p}_{i}\right)+\left[\frac{d}{d{p}_{i}}L\left(y,{p}_{i}\right)\right]{O}_{{value}}+\frac{1}{2}\left[\frac{{d}^{2}}{d{p}_{i}^{2}}L\left(y,{p}_{i}\right)\right]{O}_{{value}}^{2} \\ = \, 	 L\left(y,{p}_{i}\right)+g{O}_{{value}}+\frac{1}{2}{{hO}}_{{value}}^{2}$$

$L\left(y,{p}_{i}\right)$ is the loss function of the previous prediction, and its first and second derivative are labeled as *g* and *h*, respectively. The optimum output value could then be derived with *G* and *H* (sum of *g* and *h*) as:

$${O}_{{valuej}}=-\frac{1}{2}\mathop{\sum }\limits_{j=1}^{t}\frac{{G}_{j}^{2}}{{H}_{j}+\lambda }+\gamma T$$

The detailed mathematical model and algorithm are described in previous literature [42]. This model is able to characterize interactions and nonlinearity [21]. The tuning hyperparameters were calibrated by parallelizable Bayesian optimization based on seven initialization evaluations and multiple epochs, using the R package “ParBayesianOptimization” [43, 44]. We ran training XGboost models with 3000 rounds at first, then the optimal number of rounds (*n*) was selected by mean-squared error (MSE) as the following equation:

$${{MSE}}_{n} \, < \, 0.99* \frac{1}{20}\left({{MSE}}_{n-1}+\ldots +{{MSE}}_{n-21}\right)$$

The final XGBoost analysis was conducted with all hyperparameters using the R package “xgboost” [40]. Finally, we used the Shapley (SHAP) value to interpret and visualize the results from the XGboost machine learning model with higher transparency by the R package “SHAPforxgboost” [45, 46], and it was commonly used in previous studies [21, 22, 47]. The SHAP value unifily measures the importance of each land use exposure on GBI from the XGBoost model based on the cooperative game theory [45]. The direction of SHAP value indicates whether each land use exposure impacts positively or negatively the prediction for GBI. The XGboost model was conducted twice. First, we put all land use exposures and demographic covariates into the model, then social indicators were added.

Models were performed among overall participants and in the two clusters. We used root-mean-squared error (RMSE) to measure model performance in the training and testing subsets, which is a weighted measure calculated between forecast and observed values.

#### Sensitivity analysis

To control the potential genetic effect, we further performed the linear mixed model, in which the twin pair was assigned as the fixed term in the model. This model was to specify that the land use exposures did not vary between cotwins and to compute their within-pair effect. Two adjustment plans were employed, excluding zygosity and parental education which do not vary within pairs. Then, we conducted a post-hoc linear regression between the land use mix index and log-transformed GBI score, which aims to compare with our novel findings. Two adjustment plans were employed and the cluster effect of sampling based on families of twin pairs was controlled by the robust standard error. A *p* value less than 0.05 was considered statistically significant and 95% confidence intervals (CI) are reported.

## Results

### K-means clustering and descriptive statistics

**Fig. 1:**
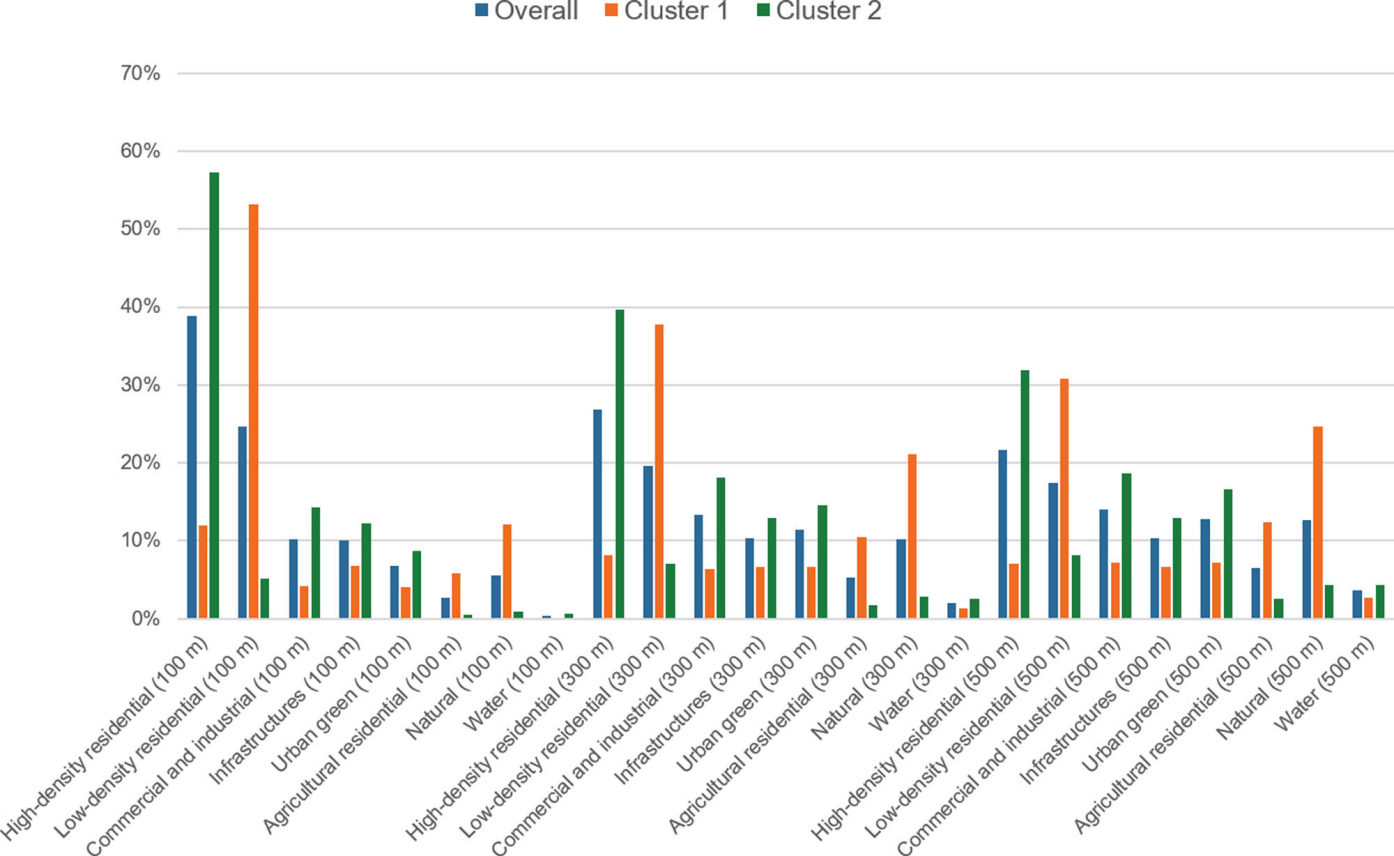
Histogram of percentage of land use exposure among overall participants, those in Cluster 1, and in Cluster 2.

Figure 1 depicts the distribution of each land use category overall and in the two clusters. Cluster 2 had a higher percentage of high-density residential land use, while Cluster 1 had a higher percentage of low-density residential land use regardless of the buffer radii of the twins’ location. Supplementary Fig. 2 shows the twins’ location in the greater Helsinki areas (as an example), and twins from Cluster 2 lived in more urbanized areas (often close to city or town centers), while twins from Cluster 1 were more suburban. Variable names and details are shown in Supplementary Table 2. We also calculated the simple ratios of means between the two clusters and found low-density residential, agricultural residential, and natural land use in a 100 m buffer have notably “relative” differences between the two clusters (ratio>10). According to the correlation matrix based on the training subset (Supplemental Fig. 3), the same land use with different radii of the buffer zone was highly correlated. High-density and low-density residential land use were negatively correlated. Notably, there was a higher number of cotwins from MZ pairs who both lived in Cluster 1 than lived discordantly, compared to DZ pairs (Supplementary Table 3).

Table 1 shows the distribution of characteristics overall and in the two clusters. Overall, the majority of twins are female (58.7%), dizygotic (61.3%), and reported never smoking (55.1%) in the young adulthood questionnaire. Additionally, 48.8% and 67.7% of twins reported that they were in full-time work and had attended senior high school, respectively. The majority (51.1%) of twins’ parents had limited education levels (less than senior high school). The means of GBI score were 4.4, 4.1, and 4.7 among overall participants, those in Cluster 1 (suburban), and in Cluster 2 (city center), respectively, and their distributions are presented in Supplementary Fig. 4. Unsupervised K-means clustering did not take into account these demographic covariates. We observed significant differences in smoking, working status, secondary level school, and parental education between the two clusters by Chi-squared test or univariable linear regression accounting for twin sampling. There were more twins who currently smorked, worked full time, and attended vocational schools in Cluster 1 than in Cluster 2, but parents in Cluster 2 had a lower percentage of receiving limited education. Addtionally, in all four social indicators, there were significant differences between clusters.

**Table 1:** Characteristics of all included twins overall and in the two clusters. The *p* values are for differences between Clusters 1 and 2 by Chi-squared test or univariable linear regression accounting for twin sampling.

Characteristic	*N* (%) / Mean (SD)			*P* value (between clusters)
	Overall (individual twin *n* = 1804)	Cluster 1 (individual twin *n* = 736)	Cluster 2 (individual twin *n* = 1068)	
GBI in young adulthood	4.42 (4.7)	4.05 (4.4)	4.67 (4.8)	0.01
*Demographic covariates*				
Sex				0.16
Male	745 (41.3)	289 (39.3)	456 (42.7)	
Female	1059 (58.7)	447 (60.7)	612 (57.3)	
Zygosity				0.92
Monozygotic	615 (34.1)	252 (34.2)	363 (34.0)	
Dizygotic	1105 (61.3)	448 (60.9)	657 (61.5)	
Unknown	84 (4.7)	36 (4.9)	48 (4.5)	
Smoking				
Never	994 (55.1)	405 (55)	589 (55.2)	0.03
Former	191 (10.6)	78 (10.6)	113 (10.6)	
Occasional	205 (11.4)	66 (9.0)	139 (13.0)	
Current	414 (23.0)	187 (25.4)	227 (21.3)	
Work				<0.0001
Full-time work	880 (48.8)	409 (55.6)	471 (44.1)	
Part-time work	280 (15.5)	94 (12.8)	186 (17.4)	
Irregular work	239 (13.3)	76 (10.3)	163 (15.3)	
Not working	405 (22.5)	157 (21.3)	248 (23.2)	
Secondary level school				<0.0001
Vocational	486 (26.9)	262 (35.6)	224 (21.0)	
Senior high school	1222 (67.7)	439 (59.7)	783 (73.3)	
None	96 (5.3)	35 (4.8)	61 (5.7)	
Parental education				<0.0001
Limited	922 (51.1)	429 (58.3)	493 (46.2)	
Intermediate	410 (22.7)	155 (21.1)	255 (23.9)	
High	472 (26.2)	152 (20.7)	320 (30.0)	
Age	24.07 (1.7)	24.15 (1.7)	24.01 (1.7)	0.10
*Social indicators*^a^				
Age structure (%)	82.7 (7.2)	78.2 (5.8)	85.8 (6.4)	<0.0001
Education level (%)	25.8 (9.0)	21.8 (8.0)	28.5 (8.6)	<0.0001
Unemployment (%)	9.6 (4.0)	8.9 (4.1)	10.0 (3.9)	<0.0001
Income level (%)	25.5 (10.0)	26.3 (10.3)	24.9 (9.8)	0.01

^a^The detailed description of social indicators was introduced in the “Subjects and Methods” section.

### Linear elastic net regression model

After minimal adjustment of demographic covariates, in Cluster 1 (suburban), 11 land use exposures were significant enough to be captured by the linear elastic net regression model in assessing their relationship with GBI (Table 2). The agricultural residential land use in a 100 m buffer increased log-transformed GBI scores with the largest penalized coefficient (coefficient: 0.097). After further adjustment with the social indicators, the number of selected land use exposures increased to 17, and the new exposures were: urban green and natural land use in both 100 and 500 m buffers, and high-density residential and water land use in a 300 m buffer. The penalized coefficient of the agricultural residential land use in a 100 m buffer was attenuated (coefficient: 0.067), while it still had the largest effect size and was positively correlated with GBI. Surprisingly, there were no land use exposures remaining in the Cluster 2 (city center) model in neither adjustment phase. Supplemental Table 4 presents the results in the overall model, and after further adjustment, there were also more land use exposures selected. The pattern of coefficients including the effect size and direction was relatively heterogeneous with Cluster 1. The coefficients for low-density residential land use in a 100 m buffer were the same (coefficient: −0.011) between the overall and Cluster 1 models after minimal adjustment.

**Table 2:** Multiple-exposure elastic net penalized regression for associations between land use and GBI in Clusters 1 and 2. The remaining coefficients were significant enough to be selected.

Land use (Buffer) unit: %	Standardized elastic net coefficient			
	Cluster 1		Cluster 2	
	Minimally adjusted^a^	Further adjusted^b^	Minimally adjusted^a^	Further adjusted^b^
High-density residential (100 m)	0.089	0.056		
Low-density residential (100 m)	−0.011	−0.043		
Commercial and industrial (100 m)				
Infrastructures (100 m)				
Urban green (100 m)		0.001		
Agricultural residential (100 m)	0.097	0.067		
Natural (100 m)		−0.003		
Water (100 m)				
High-density residential (300 m)		0.002		
Low-density residential (300 m)				
Commercial and industrial (300 m)	0.084	0.065		
Infrastructures (300 m)	−0.031	−0.029		
Urban green (300 m)	0.081	0.058		
Agricultural residential (300 m)				
Natural (300 m)		−0.014		
Water (300 m)		0.002		
High-density residential (500 m)	0.046	0.026		
Low-density residential (500 m)	0.035	0.036		
Commercial and industrial (500 m)				
Infrastructures (500 m)	−0.012	−0.005		
Urban green (500 m)		0.010		
Agricultural residential (500 m)	−0.067	−0.019		
Natural (500 m)				
Water (500 m)	0.020	0.012		
Model feature (10-fold CV selection)	*α* = 1.00, *λ* = 0.01, Out-of-sample *R*^2^ = 0.06, CV prediction error = 0.74	*α* = 0.10, *λ* = 0.05, Out-of-sample *R*^2^ = 0.06, CV prediction error = 0.74	*α* = 1.00, *λ* = 0.04, Out-of-sample *R*^2^ = 0.10, CV prediction error=0.70	*α* = 1.00, *λ* = 0.04, Out-of-sample *R*^2^ = 0.09, CV prediction error = 0.70

^a^Adjusted for sex, zygosity, smoking, work status, secondary level school, parental education, and age when twins provided the GBI assessment in young adulthood.

^b^Adjusted for sex, zygosity, smoking, work status, secondary level school, parental education, age when twins provided the GBI assessment in young adulthood, as well as age structure, education level, unemployment, and income level.

### Refitting to linear mixed model

According to the selected land use exposures from the aforementioned elastic net regression, we refitted them into linear mixed models to assess their within-pair effect on log-transformed GBI scores (Supplementary Table 5). In Cluster 1, after minimal adjustment, commercial and industrial land use in a 300 m buffer were significantly and positively associated with GBI, while the effect attenuated after further adjustment. In the overall model, after both minimal and further adjustment, higher low-density residential land use in a 100 m buffer significantly reduced the GBI.

### XGBoost model

**Fig. 2:**
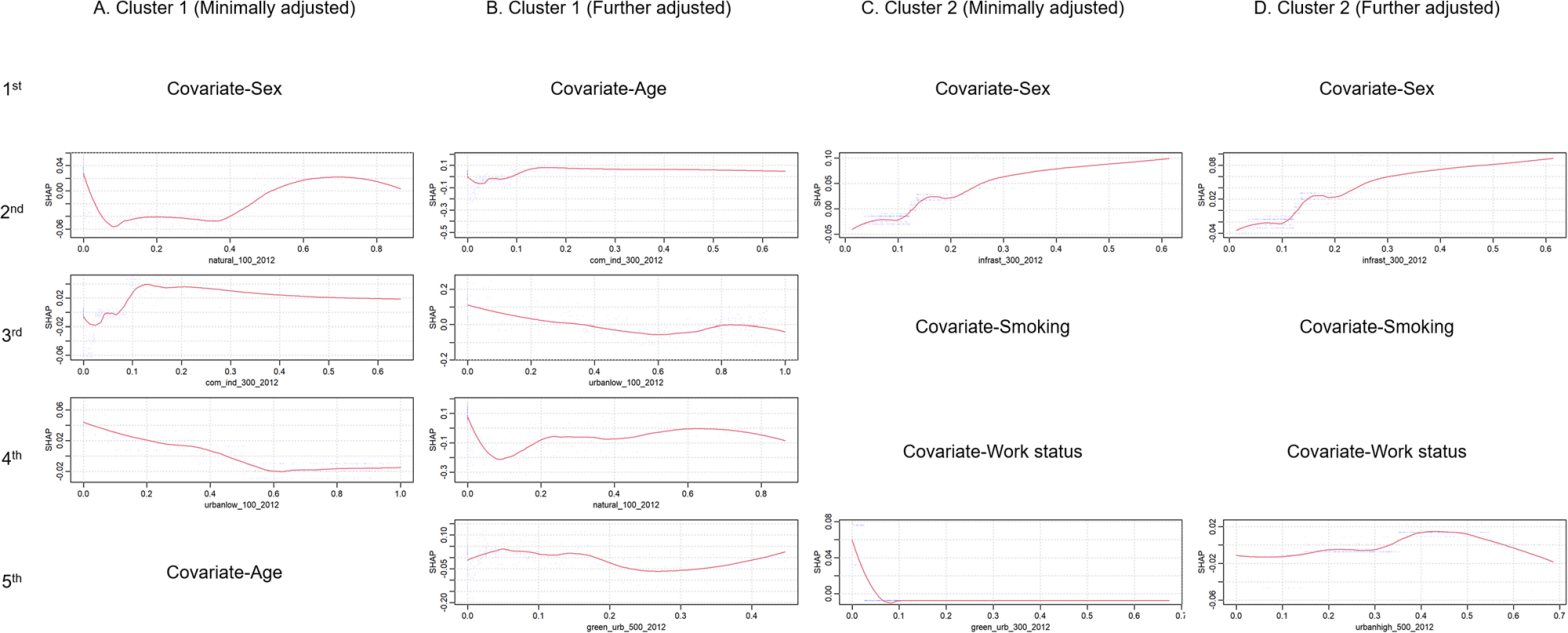
Shapley (SHAP) dependence plots of the top five most influential exposures in XGBoost models. The dependence plot shows the relationship between the SHAP value and land use exposures in four models. Cluster 1 with minimal adjustment (**A**), Cluster 1 with further adjustment (**B**), Cluster 2 with minimal adjustment (**C**), Cluster 2 with further adjustment (**D**). Demographic covariates and social indicators were included in the models but suppressed in plots to highlight land use exposures.

We listed the top five most important factors with SHAP values in each cluster’s XGBoost model. After minimal adjustment (Fig. 2A), in Cluster 1 (suburban), the most important land use exposure was natural land use in a 100 m buffer, and the second was commercial and industrial land use in a 300 m buffer. After further adjustment, natural land use in a 100 m buffer became the most important (Fig. 2B). In Cluster 2 (city center), the most important land use exposure was always infrastructure land use in a 300 m buffer after minimal (Fig. 2C) and further adjustment (Fig. 2D). Covariates were not listed and are not shown in the figure. The curve of SHAP values suggested nonlinear attribution of each land use exposure on GBI. Notablely, the curves of infrastructure land use in a 300 m buffer with SHAP values are also similar after minimal or further adjustment. There was a flat incline of SHAP value between 0 and ~10%. Then, the value sharply increased when its percentage passed ~10% and the impact of infrastructure land use in a 300 m buffer on the prediction for GBI switched from negative to positive. After the percentage was greater than ~20%, the curve slowly increased. The results of overall XGBoost models are presented in Supplemental Fig. 5. After minimal adjustment, same as Cluster 2, the most important land use exposure is infrastructure land use, but, in a 100 m buffer (Supplementary Fig. 5A). After further adjustment, the most important becames natural land use in a 100 m buffer (Supplementary Fig. 5B).

### Model performance and comparison

The standard deviations (SD) of the log-transformed GBI score were 0.8825, 0.8851, and 0.8774 among overall, Cluster 1’s and Cluster 2’s twins, respectively. The training and testing RMSE are shown in Supplementary Table 6. There are no major differences between the two types of models and clusters, and they mostly have lower SDs than those of the log-transformed GBI score, implying good model performance.

### Linear regression with the land use mix index

The results of linear regression in the overall and the two separated cluster models are presented in Table 3. In the crude Cluster 1 (suburban) model, a higher land use mix index in a 300 m buffer was significantly associated with higher log-transformed GBI scores (beta: 0.51, 95% CI: 0.02, 1.01). After either minimal or further adjustment, there was no significant association, which implies the need for complex assessments between land use and GBI.

**Table 3:** Linear regression between land use mix index and GBI in young adulthood.

Land use mix index	Log-transformed GBI scores in young adulthood			
	Mean (SD)	Unadjusted beta (95% CI)	Minimally adjusted beta (95% CI)^a^	Further adjusted beta (95% CI)^b^
*Overall (individual twin n* = *1804)*				
In a 100 m buffer	0.38 (0.14)	0.11 (−0.17, 0.39)	0.17 (−0.10, 0.44)	0.17 (−0.10, 0.44)
In a 300 m buffer	0.60 (0.12)	0.17 (−0.18, 0.52)	0.14 (−0.19, 0.48)	0.12 (−0.22, 0.45)
In a 500 m buffer	0.67 (0.11)	0.30 (−0.08, 0.68)	0.25 (−0.11, 0.62)	0.19 (−0.18, 0.56)
*Cluster 1 (individual twin n* = *736)*				
In a 100 m buffer	0.38 (0.15)	0.02 (−0.39, 0.44)	0.12 (−0.28, 0.52)	0.11 (−0.29, 0.50)
In a 300 m buffer	0.59 (0.13)	0.51 (0.02, 1.01)*	0.46 (−0.01, 0.92)	0.26 (−0.21, 0.72)
In a 500 m buffer	0.66 (0.13)	0.50 (−0.00, 1.01)	0.42 (−0.07, 0.91)	0.16 (−0.35, 0.67)
*Cluster 2 (individual twin n* = *1068)*				
In a 100 m buffer	0.38 (0.14)	0.16 (−0.21, 0.53)	0.18 (−0.18, 0.54)	0.16 (−0.21, 0.52)
In a 300 m buffer	0.60 (0.11)	−0.25 (−0.73, 0.23)	−0.27 (−0.73. 0.19)	−0.30 (−0.77, 0.18)
In a 500 m buffer	0.68 (0.10)	−0.08 (−0.63, 0.48)	−0.14 (−0.67, 0.40)	−0.19 (−0.76, 0.38)

^a^Adjusted for sex, zygosity, smoking, work status, secondary level school, parental education, and age when twins provided the GBI assessment in young adulthood.

^b^Adjusted for sex, zygosity, smoking, work status, secondary level school, parental education, age when twins provided the GBI assessment in young adulthood, as well as age structure, education level, unemployment, and income level.

*P value < 0.05.

## Discussion

Based on 1804 twins from the FinnTwin12 study with information on residential geocodes linked to land use characteristics, we identified two clusters of the land use environment the twins lived. Strengthened by multiple statistical approaches, both linear and nonlinear relationships between land use and depressive symptoms were discovered to exist. In the linear elastic net penalized regression model, among overall twins and Cluster 1 (suburban)’s twins, there was a heterogeneous pattern in selected features, effect sizes, and effect directions. In the Cluster 1 model, agricultural residential land use in a 100 m buffer was associated with depressive symptoms with the largest relative effect size. After controlling for the influence of the social environment, more land use exposures were found to be associated with depressive symptoms. With further control of the genetic effect, based on the refitting mixed models, no land use exposure was strongly associated with depressive symptoms, implying a potential inheritable effect behind. In contrast, no land use exposures were significant enough to be attributed to depressive symptoms in Cluster 2, no matter the adjustment for the social environment, which was typical of city or town centers. The XGBoost model offered a profound understanding of the multifaceted relationships regarding the intricate interplay between various land use measures and their relative importance on depressive symptoms. The importance ranks and nonlinearity of land use exposures on depressive symptoms were heterogeneous between the overall, Cluster 1, and Cluster 2 models. The most important were commercial and industrial land use in a 300 m buffer in Cluster 1 and infrastructure land use in a 300 m buffer in Cluster 2, after adding social indicators in. As a hypothesis-generating study, elements such as population heterogeneity, environmental interaction, and characteristics of the effect (such as linearity) should be considered more in future analyses between land use, as well as the broad urban environment, and depressive symptoms.

First, the clustering analysis revealed a specific pattern in urbanization, and twins from Clusters 1 and 2 mostly lived in the “suburbs” and “city or town centers”, respectively. The land use exposures are less important to depressive symptoms among people living in city or town centers. The possible mechanisms may be through differential healthcare service access, social needs, transportation connectedness, or neighborhood environment [17, 48, 49]. For example, living in the suburbs usually requires longer house-to-job commuting distances, which has been found to be associated with poorer mental health [48]. Longer job commutes impliy greater need for transportation infrastructire, and, similar to our linear elastic net regression model, the higher percentage of infrastructure land use was related to fewer depressive symptoms in Cluster 1 (suburban). Nevertheless, Pelgrims et al. detected no significant association, after full adjustment, between green surrounding, street corridor and canyon effects, and depressive disorder among participants living in the highly urbanized Brussels, Belgium [50]. Furthermore, the impact of the social environment on the relationship between land use exposures and depressive symptoms is more pronounced in suburban areas compared to city centers. In China, the mediating role of neighborhood-level social capital was shown to be evident in the connection between urbanization and depressive symptoms [51]. Since this is a single-country study, Finland, compared to other developed countries, has quieter and greener urban spaces that need to be considered in the interpretation. We did not intend to distinguish people with an arbitrary binary classification, instead, we promote the hypothesis that the relationship between land use and depressive symptoms exists in the specific land use context.

More broadly, land use exposures, that signaled urbanization, were either selected by the penalized model or were among the top five in the XGBoost model, indicating them as good candidates to explain depressive symptoms. Niu et al. developed a framework for the coupling coordination relationship between urbanization and land use transition in China and suggested a convergence phenomenon between them [52]. Nevertheless, previous evidence on the effect of urbanization on depression is not consistent. A 2020 review found a protective effect of urbanization on depression in three Chinese studies, while four other countries’ studies had opposite findings due to different geographic regions and income levels [53]. An increasing trend in depression prevalence among young adults and those who lived in rural areas with low population density was observed in a longitudinal Germany nationwide survey [54]. However, Morozov indicated that urbanization adversely affected mental health via several factors including noise and visual aggressiveness of the environment in Russia [55]. There may be conjunct or nonadditive relationships within land use or broad urban living environments. The environMENTAL Consortium has sketched the multiple mechanisms between urban living environmental profiles with more than a hundred variables and psychiatric symptoms [12]. A typical example of complexity is the urban heat island effect, a higher regional temperature in urban areas than in surrounding rural areas. It is differentially influenced by many land use factors, in which expansion of built-up area increased but water areas reduced the regional temperature [56], and moreover, the urban heat island increases the risk of depression [53]. Additionally, the directions of two negatively correlated land use exposures’ influence were not always consistent across varying buffers, thus a single exposure cannot be inferred as a risk or protective factor. Buffers provide a consideration of contextual effect, which incarnate the spatial scale for different pathways linking urban environments to health [57]. Thus, for the implication of urban planning and improvement, we advocate that policymakers recognize the intricate nature of our urban environment and adopt a perspective that encompasses it as a holistic integration, instead of a limited set of indices or indicators.

Including multiple land use exposures in a single analytic platform allows us to disentangle the individual effects and assess the complex relationships. To some extent, machine learning models allow us to adjust or consider the mutual effect between different land use exposures instead of repeated single regression models. The linear elastic net penalized regression models selected a subset of the most important land use exposures and reduced the risk of correlating and overfitting, with better performance [38]. Because we aim to reveal relationships instead of prediction, we did not refill the land use exposures to the normal regression model and the interpretation of effect size was weakened. Lenters et al. have applied this approach to prenatal chemical exposures to solve the interconnected effects of mixtures [37]. We also observed the nonlinear relationship via the interpretable SHAP visualization from XGBoost, but, like Ohanyan and colleagues’ studies, we did not straightforwardly assess the interaction due to modest effect sizes and other factors [21, 58]. Previous applications of this machine learning method improved the prediction and forecast of air quality in China [41, 59]. Ma et al. also compared the prediction accuracy between XGBoost and Lasso penalized regression models [59], while, in our study, we wished to observe the intricate effects instead of comparing accuracy, so we used RMSE, not AUC, to evaluate model performance. Another Chinese study also explored the nonlinear effect between the built and social environments and bus use among older adults [42]. In advancing the conventional regression model with limited exposures, the utility of multiple machine learning algorithms provides a preliminary sketch of the labyrinthine relationship between urban land use and depression symptoms.

Clustering analysis focused on multiple land use exposures and facilitates the segmentation of residents for tailored epidemiological assessment of the effect of land use on depressive symptoms and customizes further improvement and intervention. The differential pattern of urban land use environment was very obvious in our findings. Methodologically, clustering analysis has gained increasing attention in the field of exposure science. Tognola and colleagues clustered children in France by exposure to extremely low-frequency magnetic fields [60], and another study developed a novel workflow in clustering with multiple features including specific and general external exposomes and identified sub-populations in type-2 diabetes patients [61].

There are some limitations in our studies. First, the information on depression symptoms was obtained before 2012, so the potential causality and direction are unable to be confirmed due to temporality. Additionally, temporality also leads to the question of the length and stability of exposures, so a lifecourse study is needed. Second, compared to previous similar studies, the sample size is relatively small. Although the two machine learning methods are able to shrink the overfitting due to the small sample size, we still need to be cautious about the findings. Third, we did not “fully” leverage the twin structure to quantify the potential genetic influence, although concordance and discordance in clusters differed between monozygotic and dizygotic twins. Instead, we used a mixed model to further explore the within-pair effect to properly control the underlying genetic effect. Incorporation of a twin design could guide the investigation of underlying genetic influence in the high-dimensional environmental study in the future. Fourth, there are potential confounding effects stemming from other physical exposures such as air pollution and noise. Although the land use exposures already carry some information about these exposures [62], our forthcoming endeavors will employ advanced techniques and models to measure these. Finally, the interpretability of the machine learning model is a significant challenge that required more endeavor in the field of data science. We found the nonlinearity pattern, but it is difficult to elaborate on. This study is a pilot study for exploration, and further follow-up studies are welcome to strengthen the evidence.

## Conclusion

This study is the first, to our knowledge, to investigate the complex relationship between multiple urban land use exposures and depressive symptoms in young adulthood. The pluralistic multi-model inferences selected or prioritized the more important urban land use exposures to depressive symptoms and revealed linear and nonlinear relationships, which advances the conventional assessment with a single index. Clustering analysis showed a notable heterogeneous pattern in these relationships between participants with different land use environments, implying the effects are under a specific context. Due to sample size, model characteristics, and temporality, our finding interpretation is cautious at present, and more efforts are warranted to corroborate.

## Supplementary information

Supplemental material

## Data Availability

The FinnTwin12 data is not publicly available due to the restrictions of informed consent. However, the FinnTwin12 data is available through the Institute for Molecular Medicine Finland (FIMM) Data Access Committee (DAC) (fimm-dac@helsinki.fi) for authorized researchers who have IRB/ethics approval and an institutionally approved study plan. To ensure the protection of privacy and compliance with national data protection legislation, a data use/transfer agreement is needed, the content and specific clauses of which will depend on the nature of the requested data.

## References

[1] Twenge JM, Cooper AB, Joiner TE, Duffy ME, Binau SG. Age, period, and cohort trends in mood disorder indicators and suicide-related outcomes in a nationally representative dataset, 2005-2017. J Abnorm Psychol. 2019;128:185–99. 30869927 10.1037/abn0000410

[2] Filatova S, Upadhyaya S, Kronström K, Suominen A, Chudal R, Luntamo T, et al. Time trends in the incidence of diagnosed depression among people aged 5–25 years living in Finland 1995–2012. Nord J Psychiatry. 2019;73:475–81. 31443615 10.1080/08039488.2019.1652342

[3] Shorey S, Ng ED, Wong CHJ. Global prevalence of depression and elevated depressive symptoms among adolescents: a systematic review and meta-analysis. Br J Clin Psychol. 2022;61:287–305. 34569066 10.1111/bjc.12333

[4] Wang C, Wen W, Zhang H, Ni J, Jiang J, Cheng Y, et al. Anxiety, depression, and stress prevalence among college students during the COVID-19 pandemic: a systematic review and meta-analysis. *J Am Coll Heal.* 2021;71:2123–30. 10.1080/07448481.2021.1960849 34469261

[5] Hawes MT, Szenczy AK, Klein DN, Hajcak G, Nelson BD. Increases in depression and anxiety symptoms in adolescents and young adults during the COVID-19 pandemic. Psychol Med. 2022;52:3222–30. 33436120 10.1017/S0033291720005358 PMC7844180

[6] Pozuelo JR, Desborough L, Stein A, Cipriani A. Systematic review and meta-analysis: depressive symptoms and risky behaviors among adolescents in low- and middle-income countries. J Am Acad Child Adolesc Psychiatry. 2022;61:255–76. 34015483 10.1016/j.jaac.2021.05.005

[7] Sullivan PF, Neale MC, Kendler KS. Genetic epidemiology of major depression: review and meta-analysis. Am J Psychiatry. 2000;157:1552–62. 11007705 10.1176/appi.ajp.157.10.1552

[8] Lau JYF, Eley TC. Changes in genetic and environmental influences on depressive symptoms across adolescence and young adulthood. Br J Psychiatry. 2006;189:422–7. 17077432 10.1192/bjp.bp.105.018721

[9] Hur Y-M. Sex differences in genetic and environmental contributions to depression symptoms in South Korean adolescent and young adult twins. Twin Res Hum Genet. 2008;11:306–13. 18498208 10.1375/twin.11.3.306

[10] Nuissl H, Siedentop S. Urbanisation and land use change BT - sustainable land management in a European context: a co-design approach. In: Weith T, Barkmann T, Gaasch N, Rogga S, Strauß C, Zscheischler J (eds). Springer International Publishing: Cham, 2021, pp 75–99.

[11] Dong G, Ge Y, Jia H, Sun C, Pan S. Land use multi-suitability, land resource scarcity and diversity of human needs: a new framework for land use conflict identification. Land. 2021;10. 10.3390/land10101003.

[12] Xu J, Liu, Polemiti N, Garcia-Mondragon E, Tang J L, Liu X, et al. Effects of urban living environments on mental health in adults. Nat Med. 2023;29:1456–67. 37322117 10.1038/s41591-023-02365-w PMC10287556

[13] Voutilainen A, Hartikainen S, Sherwood PR, Taipale H, Tolppanen A-M, Vehviläinen-Julkunen K. Associations across spatial patterns of disease incidences, socio-demographics, and land use in Finland 1991–2010. Scand J Public Health. 2015;43:356–63. 25743878 10.1177/1403494815572721

[14] Sambell CE, Holland GJ, Haslem A, Bennett AF. Diverse land-uses shape new bird communities in a changing rural region. Biodivers Conserv. 2019;28:3479–96.

[15] Brown BB, Yamada I, Smith KR, Zick CD, Kowaleski-Jones L, Fan JX. Mixed land use and walkability: variations in land use measures and relationships with BMI, overweight, and obesity. Health Place. 2009;15:1130–41. 19632875 10.1016/j.healthplace.2009.06.008 PMC2778756

[16] Miles R, Coutts C, Mohamadi A. Neighborhood urban form, social environment, and depression. J Urban Heal. 2012;89:1–18. 10.1007/s11524-011-9621-2 PMC3284588 22038283

[17] Melis G, Gelormino E, Marra G, Ferracin E, Costa G. The effects of the urban built environment on mental health: a cohort study in a large Northern Italian city. *Int J Environ Res Public Health.* 2015;12. 10.3390/ijerph121114898. 10.3390/ijerph121114898 PMC4661687 26610540

[18] Wu W, Chen WY, Yun Y, Wang F, Gong Z. Urban greenness, mixed land-use, and life satisfaction: evidence from residential locations and workplace settings in Beijing. Landsc Urban Plan. 2022;224:104428.

[19] Bordoloi R, Mote A, Sarkar PP, Mallikarjuna C. Quantification of land use diversity in the context of mixed land use. Procedia - Soc Behav Sci. 2013;104:563–72.

[20] Guloksuz S, van Os J, Rutten BPF. The exposome paradigm and the complexities of environmental research in psychiatry. JAMA Psychiatry. 2018;75:985–6. 29874362 10.1001/jamapsychiatry.2018.1211

[21] Ohanyan H, Portengen L, Huss A, Traini E, Beulens JWJ, Hoek G, et al. Machine learning approaches to characterize the obesogenic urban exposome. Environ Int. 2022;158:107015. 34991269 10.1016/j.envint.2021.107015

[22] Ohanyan H, Portengen L, Kaplani O, Huss A, Hoek G, Beulens JWJ, et al. Associations between the urban exposome and type 2 diabetes: Results from penalised regression by least absolute shrinkage and selection operator and random forest models. Environ Int. 2022;170:107592. 36306550 10.1016/j.envint.2022.107592

[23] Lydiane A, Lützen P, Marc C-H, Xavier B, Lise G-A, Valérie S, et al. A systematic comparison of linear regression–based statistical methods to assess exposome-health associations. Environ Health Perspect. 2016;124:1848–56. 27219331 10.1289/EHP172 PMC5132632

[24] Maitre L, Guimbaud J-B, Warembourg C, Güil-Oumrait N, Petrone PM, Chadeau-Hyam M, et al. State-of-the-art methods for exposure-health studies: results from the exposome data challenge event. Environ Int. 2022;168:107422. 36058017 10.1016/j.envint.2022.107422

[25] Hoskovec L, Benka-Coker W, Severson R, Magzamen S, Wilson A. Model choice for estimating the association between exposure to chemical mixtures and health outcomes: a simulation study. PLoS One. 2021;16:e0249236. 33765068 10.1371/journal.pone.0249236 PMC7993848

[26] Rose RJ, Salvatore JE, Aaltonen S, Barr PB, Bogl LH, Byers HA, et al. FinnTwin12 cohort: an updated review. Twin Res Hum Genet. 2019;22:302–11. 31640839 10.1017/thg.2019.83 PMC7108792

[27] Kokko K, Pulkkinen L. Unemployment and psychological distress: mediator effects. J Adult Dev. 1998;5:205–17.

[28] Depue RA, Slater JF, Wolfstetter-Kausch H, Klein D, Goplerud E, Farr D. A behavioral paradigm for identifying persons at risk for bipolar depressive disorder: A conceptual framework and five validation studies. J Abnorm Psychol. 1981;90:381–437. 7298991 10.1037//0021-843x.90.5.381

[29] Bucholz KK, Cadoret R, Cloninger CR, Dinwiddie SH, Hesselbrock VM, Nurnberger JI, et al. A new, semi-structured psychiatric interview for use in genetic linkage studies: a report on the reliability of the SSAGA. J Stud Alcohol. 1994;55:149–58. 8189735 10.15288/jsa.1994.55.149

[30] Urban Atlas LCLU 2012. 2021. http://land.copernicus.eu/local/urban-atlas/urban-atlas-2012/view (accessed 7 Sep 2022).

[31] Lo Papa G, Palermo V, Dazzi C. Is land-use change a cause of loss of pedodiversity? The case of the Mazzarrone study area, Sicily. Geomorphology. 2011;135:332–42.

[32] Huppertz C, Bartels M, de Geus EJC, van Beijsterveldt CEM, Rose RJ, Kaprio J, et al. The effects of parental education on exercise behavior in childhood and youth: a study in Dutch and Finnish twins. Scand J Med Sci Sports. 2017;27:1143–56. 27455885 10.1111/sms.12727 PMC5266726

[33] Kantardzic M. Data mining: concepts, models, methods, and algorithms. John Wiley & Sons, Inc., 2019 https://www.wiley.com/en-us/Data+Mining%3A+Concepts%2C+Models%2C+Methods%2C+and+Algorithms%2C+3rd+Edition-p-9781119516071.

[34] Liao M, Li Y, Kianifard F, Obi E, Arcona S. Cluster analysis and its application to healthcare claims data: a study of end-stage renal disease patients who initiated hemodialysis. BMC Nephrol. 2016;17:25. 26936756 10.1186/s12882-016-0238-2 PMC4776444

[35] Kassambara A. Practical guide to cluster analysis in R. 1st ed. STHDA, 2017.

[36] Rousseeuw PJ. Silhouettes: a graphical aid to the interpretation and validation of cluster analysis. J Comput Appl Math. 1987;20:53–65.

[37] Lenters V, Portengen L, Rignell-Hydbom A, Jönsson BAG, Lindh CH, Piersma AH, et al. Prenatal phthalate, perfluoroalkyl acid, and organochlorine exposures and term birth weight in three birth cohorts: multi-pollutant models based on elastic net regression. Environ Health Perspect. 2016;124:365–72. 26115335 10.1289/ehp.1408933 PMC4786980

[38] Zou H, Hastie T.Regularization and variable selection via the elastic net.J R Stat Soc Ser B (Statistical Methodol.). 2005;67:301–20.

[39] Friedman JH, Hastie T, Tibshirani R. Regularization paths for generalized linear models via coordinate descent. J Stat Softw. 2010;33:1–22. 20808728 PMC2929880

[40] Chen T, Guestrin C. XGBoost: a scalable tree boosting system. *Proc 22nd ACM SIGKDD Int Conf Knowl Discov Data Min* 2016. 10.1145/2939672.

[41] Chen Z-Y, Zhang T-H, Zhang R, Zhu Z-M, Yang J, Chen P-Y, et al. Extreme gradient boosting model to estimate PM2.5 concentrations with missing-filled satellite data in China. Atmos Environ. 2019;202:180–9.

[42] Wang L, Zhao C, Liu X, Chen X, Li C, Wang T, et al. Non-linear effects of the built environment and social environment on bus use among older adults in China: an application of the XGBoost model. *Int J Environ Res Public Health.* 2021;18. 10.3390/ijerph18189592. 10.3390/ijerph18189592 PMC8468485 34574517

[43] Wilson S. Parallel Bayesian optimization of hyperparameters. 2022 https://cran.r-project.org/web/packages/ParBayesianOptimization/ParBayesianOptimization.pdf.

[44] Rincourt S-L, Michiels S, Drubay D. Complex disease individual molecular characterization using infinite sparse graphical independent component analysis. Cancer Inf. 2022;21:11769351221105776. 10.1177/11769351221105776 PMC9290103 35860346

[45] Lundberg S, Lee S-I. A unified approach to interpreting model predictions. 2017. 10.48550/arxiv.1705.07874.

[46] Liu Y, Just A, Mayer M. SHAPforxgboost: SHAP plots for “XGBoost”. *R Packag version 010* 2020.

[47] Chen Y, Zhang X, Grekousis G, Huang Y, Hua F, Pan Z, et al. Examining the importance of built and natural environment factors in predicting self-rated health in older adults: an extreme gradient boosting (XGBoost) approach. J Clean Prod. 2023;413:137432.

[48] Shen Y, Ta N, Liu Z. Job-housing distance, neighborhood environment, and mental health in suburban Shanghai: a gender difference perspective. Cities. 2021;115:103214.

[49] McLoughlin C, McLoughlin A, Jain S, Abdalla A, Cooney J, MacHale S. The suburban-city divide: an evaluation of emergency department mental health presentations across two centres. Ir J Med Sci. 2021;190:1523–8. 33392979 10.1007/s11845-020-02496-w

[50] Pelgrims I, Devleesschauwer B, Guyot M, Keune H, Nawrot TS, Remmen R, et al. Association between urban environment and mental health in Brussels, Belgium. BMC Public Health. 2021;21:635. 33794817 10.1186/s12889-021-10557-7 PMC8015067

[51] Wang R, Xue D, Liu Y, Chen H, Qiu Y. The relationship between urbanization and depression in China: the mediating role of neighborhood social capital. Int J Equity Health. 2018;17:105. 30041631 10.1186/s12939-018-0825-x PMC6056990

[52] Niu B, Ge D, Yan R, Ma Y, Sun D, Lu M, et al. The evolution of the interactive relationship between urbanization and land-use transition: a case study of the Yangtze River Delta. *Land*. 2021;10. 10.3390/land10080804.

[53] Sampson L, Ettman CK, Galea S. Urbanization, urbanicity, and depression: a review of the recent global literature. *Curr Opin Psychiatry.* 2020;33. 10.1097/YCO.0000000000000588. 10.1097/YCO.0000000000000588 32040041

[54] Steffen A, Thom J, Jacobi F, Holstiege J, Bätzing J. Trends in prevalence of depression in Germany between 2009 and 2017 based on nationwide ambulatory claims data. J Affect Disord. 2020;271:239–47. 32479322 10.1016/j.jad.2020.03.082

[55] Morozov PV. Mental health and urbanization: a Russian perspective. *Curr Opin Psychiatry.* 2018;31. 10.1097/YCO.0000000000000415. 10.1097/YCO.0000000000000415 29528899

[56] Chen X-L, Zhao H-M, Li P-X, Yin Z-Y. Remote sensing image-based analysis of the relationship between urban heat island and land use/cover changes. Remote Sens Environ. 2006;104:133–46.

[57] Markevych I, Schoierer J, Hartig T, Chudnovsky A, Hystad P, Dzhambov AM, et al. Exploring pathways linking greenspace to health: theoretical and methodological guidance. Environ Res. 2017;158:301–17. 28672128 10.1016/j.envres.2017.06.028

[58] Wright MN, Ziegler A, König IR. Do little interactions get lost in dark random forests? BMC Bioinform. 2016;17:145. 10.1186/s12859-016-0995-8 PMC4815164 27029549

[59] Ma J, Yu Z, Qu Y, Xu J, Cao Y. Application of the XGBoost machine learning method in PM2.5 prediction: a case study of Shanghai. Aerosol Air Qual Res. 2020;20:128–38.

[60] Tognola G, Bonato M, Chiaramello E, Fiocchi S, Magne I, Souques M, et al. Use of machine learning in the analysis of indoor ELF MF exposure in children. *Int J Environ Res Public Health.* 2019;16. 10.3390/ijerph16071230. 10.3390/ijerph16071230 PMC6479449 30959870

[61] Bej S, Sarkar J, Biswas S, Mitra P, Chakrabarti P, Wolkenhauer O. Identification and epidemiological characterization of type-2 diabetes sub-population using an unsupervised machine learning approach. Nutr Diabetes. 2022;12:27. 35624098 10.1038/s41387-022-00206-2 PMC9142500

[62] Azmi WNFW, Pillai TR, Latif MT, Koshy S, Shaharudin R. Application of land use regression model to assess outdoor air pollution exposure: a review. Environ Adv. 2023;11:100353.

