# Supplementary material for: The association between urban land use and depressive symptoms in young adulthood: a FinnTwin12 cohort study
Source: J Expo Sci Environ Epidemiol. 2023 Dec 11;34(5):770–9. doi: 10.1038/s41370-023-00619-w (PMC11446816; doi:10.1038/s41370-023-00619-w)
Supplement: Supplementary file 1 — Supplemental material [file 41370_2023_619_MOESM1_ESM.docx]

Title: The association between urban land use and depressive symptoms in young adulthood: a FinnTwin12 cohort study

Zhiyang Wang et al. – Online supplemental material

Abstract for supplemental material

Supplemental Tables 1 and 2 detail subsets’ characteristics and land use exposure, respectively. Supplemental Table 3 describes whether cotwins lived discordantly in clusters. Supplemental Table 4 shows the results of elastic net penalized regression among overall participants. Supplemental Table 5 shows the results of refitted linear mixed models between selected land use exposures and GBI. Supplemental Table 6 showed models’ performance. Supplemental Figure 1 shows the results of the K-means clustering. Supplemental Figure 2 maps twins in the greater Helsinki area in 2012. Supplemental Figure 3 is the correlation matrix between land use exposures. Supplemental Figure 4 presents the distribution of the GBI score. Supplemental Figure 5 shows the results of XGBoost models among overall participants.

Supplemental Table 1: Characteristics of training and testing subsets

| **Characteristic** | **N (%) / Mean (SD)** | |
| --- | --- | --- |
|  | Training  (individual twin n=1215) | Testing (individual twin n=589) |
| **GBI in young adulthood** | 4.49 (4.7) | 4.28 (4.6) |
| *Demographic covariates* |  |  |
| **Sex** |  |  |
| Male | 508 (41.8) | 237 (40.2) |
| Female | 707 (58.2) | 352 (59.8) |
| **Zygosity** |  |  |
| Monozygotic | 392 (32.3) | 223 (37.9) |
| Dizygotic | 763 (62.8) | 342 (58.1) |
| Unknown | 60 (4.9) | 24 (4.1) |
| **Smoking** |  |  |
| Never | 654 (53.8) | 340 (57.7) |
| Quit | 137 (11.3) | 54 (9.2) |
| Occasional | 149 (12.3) | 56 (9.5) |
| Current | 275 (22.6) | 139 (23.6) |
| **Work** |  |  |
| Full-time work | 606 (49.9) | 274 (46.5) |
| Part-time work | 166 (13.7) | 114 (19.4) |
| Irregular work | 159 (13.1) | 80 (13.6) |
| Not working | 284 (23.4) | 121 (20.5) |
| **Secondary level school** |  |  |
| Vocational | 332 (27.3) | 154 (26.2) |
| Senior high school | 821 (67.6) | 401 (68.1) |
| None | 62 (5.1) | 34 (5.8) |
| **Parental education** |  |  |
| Limited | 644 (53.0) | 278 (47.2) |
| Intermediate | 271 (22.3) | 139 (23.6) |
| High | 300 (24.7) | 172 (29.2) |
| **Age** | 24.14 (1.7) | 23.92 (1.7) |
| *Social indicators* ^a^ |  |  |
| **Age structure (%)** | 82.6 (7.2) | 83.0 (7.0) |
| **Education level (%)** | 25.8 (9.0) | 25.8 (9.1) |
| **Unemployment (%)** | 9.5 (4.0) | 9.6 (4.1) |
| **Income level (%)** | 25.5 (10.0) | 25.4 (10.1) |

^a^ The detailed description of social indicators was introduced in the Subjects and Methods

section.

Supplemental Table 2: Land use characteristics of overall twins and in the two clusters

| Land use (Buffer) unit: % | Variable name | mean ± SD | | | Ratio of means  between two clusters ^a^ |
| --- | --- | --- | --- | --- | --- |
|  |  | Overall  (individual twin n=1804) | Cluster 1  (individual twin n=736) | Cluster 2  (individual twin n=1068) |  |
| High-density residential (100 m) | urbanhigh_100_2012 | 0.388 (0.330) | 0.120 (0.217) | 0.573 (0.261) | 4.78 |
| Low-density residential (100 m) | urbanlow_100_2012 | 0.247 (0.324) | 0.532 (0.311) | 0.051 (0.130) | 10.43 |
| Commercial and industrial (100 m) | com_ind_100_2012 | 0.102 (0.192) | 0.042 (0.113) | 0.143 (0.222) | 3.40 |
| Infrastructures (100 m) | infrast_100_2012 | 0.100 (0.078) | 0.068 (0.044) | 0.122 (0.089) | 1.79 |
| Urban green (100 m) | green_urb_100_2012 | 0.068 (0.116) | 0.040 (0.089) | 0.087 (0.129) | 2.18 |
| Agricultural residential (100 m) | agric_100_2012 | 0.027 (0.094) | 0.058 (0.136) | 0.005 (0.033) | 11.60 |
| Natural (100 m) | natural_100_2012 | 0.055 (0.133) | 0.121 (0.181) | 0.009 (0.045) | 13.44 |
| Water (100 m) | water_100_2012 | 0.004 (0.028) | 0.001 (0.010) | 0.006 (0.036) | 6.00 |
| High-density residential (300 m) | urbanhigh_300_2012 | 0.268 (0.217) | 0.082 (0.103) | 0.396 (0.178) | 4.83 |
| Low-density residential (300 m) | urbanlow_300_2012 | 0.196 (0.214) | 0.377 (0.202) | 0.070 (0.103) | 5.39 |
| Commercial and industrial (300 m) | com_ind_300_2012 | 0.133 (0.141) | 0.064 (0.090) | 0.181 (0.150) | 2.83 |
| Infrastructures (300 m) | infrast_300_2012 | 0.103 (0.066) | 0.066 (0.037) | 0.129 (0.069) | 1.95 |
| Urban green (300 m) | green_urb_300_2012 | 0.114 (0.114) | 0.067 (0.086) | 0.146 (0.119) | 2.18 |
| Agricultural residential (300 m) | agric_300_2012 | 0.053 (0.120) | 0.105 (0.164) | 0.018 (0.053) | 5.83 |
| Natural (300 m) | natural_300_2012 | 0.102 (0.163) | 0.211 (0.197) | 0.028 (0.068) | 7.54 |
| Water (300 m) | water_300_2012 | 0.020 (0.062) | 0.013 (0.047) | 0.025 (0.070) | 1.92 |
| High density residential (500 m) | urbanhigh_500_2012 | 0.217 (0.173) | 0.070 (0.077) | 0.319 (0.146) | 4.56 |
| Low density residential (500 m) | urbanlow_500_2012 | 0.174 (0.171) | 0.308 (0.165) | 0.082 (0.098) | 3.76 |
| Commercial and industrial (500 m) | com_ind_500_2012 | 0.140 (0.121) | 0.072 (0.081) | 0.187 (0.121) | 2.60 |
| Infrastructures (500 m) | infrast_500_2012 | 0.103 (0.063) | 0.066 (0.040) | 0.129 (0.064) | 1.95 |
| Urban green (500 m) | green_urb_500_2012 | 0.128 (0.108) | 0.072 (0.084) | 0.166 (0.106) | 2.31 |
| Agricultural residential (500 m) | agric_500_2012 | 0.065 (0.123) | 0.124 (0.162) | 0.025 (0.059) | 4.96 |
| Natural (500 m) | natural_500_2012 | 0.126 (0.169) | 0.246 (0.190) | 0.043 (0.081) | 5.72 |
| Water (500 m) | water_500_2012 | 0.037 (0.081) | 0.027 (0.068) | 0.043 (0.089) | 1.59 |

^a^ The larger of the means is used in the numerator in the ratio**.**

Supplemental Table 3: Cotwin concordance and discordance in clusters

| **Zygosity  (Twin pair n=589)** | **N. (%)** | | |
| --- | --- | --- | --- |
|  | Both in Cluster 1 | Both in Cluster 2 | Discordant in clusters |
| Monozygotic | 116 (26.0) | 194 (43.5) | 136 (30.5) |
| Dizygotic | 132 (19.3) | 282 (41.2) | 270 (39.5) |
| Unknown | 10 (20.8) | 14 (29.2) | 24 (50.0) |

Supplemental Table 4: Multiple exposure elastic net penalized regression for associations between land use and GBI among overall participants. The remaining coefficients shown here were significant enough to be selected.

| Land use (Buffer) unit: % | Standardized elastic net coefficient | |
| --- | --- | --- |
|  | Minimally adjusted ^a^ | Further adjusted ^b^ |
| High-density residential (100m) |  |  |
| Low-density residential (100m) | 0.011 | 0.005 |
| Commercial and industrial (100m) |  |  |
| Infrastructures (100m) |  | 0.025 |
| Urban green (100m) |  |  |
| Agricultural residential (100m) |  | 0.020 |
| Natural (100m) | 0.020 |  |
| Water (100m) |  | 0.017 |
| High-density residential (300m) | 0.013 | 0.004 |
| Low-density residential (300m) |  |  |
| Commercial and industrial (300m) |  |  |
| Infrastructures (300m) | 0.003 | 0.013 |
| Urban green (300m) |  |  |
| Agricultural residential (300m) |  |  |
| Natural (300m) | 0.009 | 0.024 |
| Water (300m) |  |  |
| High-density residential (500m) | 0.002 | 0.006 |
| Low-density residential (500m) |  |  |
| Commercial and industrial (500m) |  |  |
| Infrastructures (500m) |  | 0.008 |
| Urban green (500m) |  |  |
| Agricultural residential (500m) |  |  |
| Natural (500m) |  |  |
| Water (500m) |  |  |
| Model feature (10 fold CV selection) | α=0.10, λ =0.25, Out of sample R^2^=0.09, CV prediction error=0.73 | α=0.10, λ =0.11, Out of sample R^2^=0.10, CV prediction error=0.70 |

^a^ Adjusted for sex, zygosity, smoking, work status, secondary level school, parental education, and age when twins provided the GBI assessment in young adulthood.

^b^ Adjusted for sex, zygosity, smoking, work status, secondary level school, parental education, age when twins provided the GBI assessment in young adulthood, as well as age structure, education level, unemployment, and income level.

Supplemental Table 5: unpenalized linear mixed models between the selected land use exposures from elastic net penalized regression and GBI.

| Land use (Buffer) unit: % | Beta (95% CI) | | | |
| --- | --- | --- | --- | --- |
|  | Overall | | Cluster 1 | |
|  | Minimally adjusted ^a^ | Further adjusted ^b^ | Minimally adjusted ^a^ | Further adjusted ^b^ |
| High-density residential (100m) |  |  | 0.61 (-0.15, 1.38) | 0.34 (-0.97, 1.66) |
| Low-density residential (100m) | -0.41 (-0.67, -0.16)* | -0.47 (-0.73, -0.21)* | -0.15 (-0.82, 0.52) | -0.04 (-1.25, 1.16) |
| Commercial and industrial (100m) |  |  |  |  |
| Infrastructures (100m) |  | 0.53 (-0.37, 1.43) |  |  |
| Urban green (100m) |  |  |  | 0.53 (-1.06, 2.12) |
| Agricultural residential (100m) |  | 0.03 (-0.69, 0.75) | -0.52 (-1.75, 0.72) | -0.46 (-2.10, 1.17) |
| Natural (100m) | 0.24 (-0.49, 0.97) |  |  | 0.42 (-1.13, 1.97) |
| Water (100m) |  | -1.49 (-3.68, 0.69) |  |  |
| High-density residential (300m) | -0.52 (-1.37, 0.33) | -0.52 (-1.36, 0.32) |  | 1.31 (-2.73, 5.34) |
| Low-density residential (300m) |  |  |  |  |
| Commercial and industrial (300m) |  |  | 2.44 (1.12, 3.75)* | 1.50 (-0.68, 3.68) |
| Infrastructures (300m) | 0.24 (-0.94, 1.41) | 0.19 (-1.92, 2.30) | -2.18 (-6.86, 2.51) | -4.44 (-9.91, 1.03) |
| Urban green (300m) |  |  | 0.08 (-1.38, 1.53) | -1.39 (-4.45, 1.67) |
| Agricultural residential (300m) |  |  |  |  |
| Natural (300m) | -0.60 (-1.26, 0.07) | -0.52 (-1.04, 0.00) |  | -1.57 (-3.73, 0.6) |
| Water (300m) |  |  |  | 2.82 (-4.19, 9.83) |
| High-density residential (500m) | -0.01 (-1.03, 1.01) | -0.07 (-1.11, 0.96) | -1.39 (-4.23, 1.44) | -3.50 (-8.15, 1.15) |
| Low-density residential (500m) |  |  | 0.41 (-0.74, 1.57) | -0.70 (-2.72, 1.32) |
| Commercial and industrial (500m) |  |  |  |  |
| Infrastructures (500m) |  | -0.56 (-2.65, 1.53) | 0.64 (-5.51, 6.80) | 0.64 (-5.39, 6.68) |
| Urban green (500m) |  |  |  | 0.39 (-2.99, 3.77) |
| Agricultural residential (500m) |  |  | -0.18 (-1.49, 1.13) | -1.38 (-3.52, 0.77) |
| Natural (500m) |  |  |  |  |
| Water (500m) |  |  | 0.75 (-1.17, 2.68) | -1.64 (-5.86, 2.58) |

^a^ Adjusted for sex, smoking, work status, secondary level school, and age when twins provided the GBI assessment in young adulthood.

^b^ Adjusted for sex, smoking, work status, secondary level school, age when twins provided the GBI assessment in young adulthood, as well as age structure, education level, unemployment, and income level.

* *P-value <0.05*

Supplemental Table 6: Model performance via root mean squared error (RMSE) for linear elastic net penalized regression and XGBoost models

| Model performance | | Minimally adjusted | | Further adjusted | |
| --- | --- | --- | --- | --- | --- |
|  |  | Training RMSE | Testing RMSE | Training RMSE | Testing RMSE |
| Linear elastic net penalized regression | Overall | 0.840 | 0.817 | 0.820 | 0.853 |
|  | Cluster 1 | 0.825 | 0.817 | 0.818 | 0.814 |
|  | Cluster 2 | 0.835 | 0.782 | 0.818 | 0.816 |
| XGBoost | Overall | 0.687 | 0.736 | 0.876 | 0.804 |
|  | Cluster 1 | 0.847 | 0.763 | 0.848 | 0.765 |
|  | Cluster 2 | 0.850 | 0.813 | 0.849 | 0.814 |

Supplemental Figure 1: 2D scatter plot of K means clustering


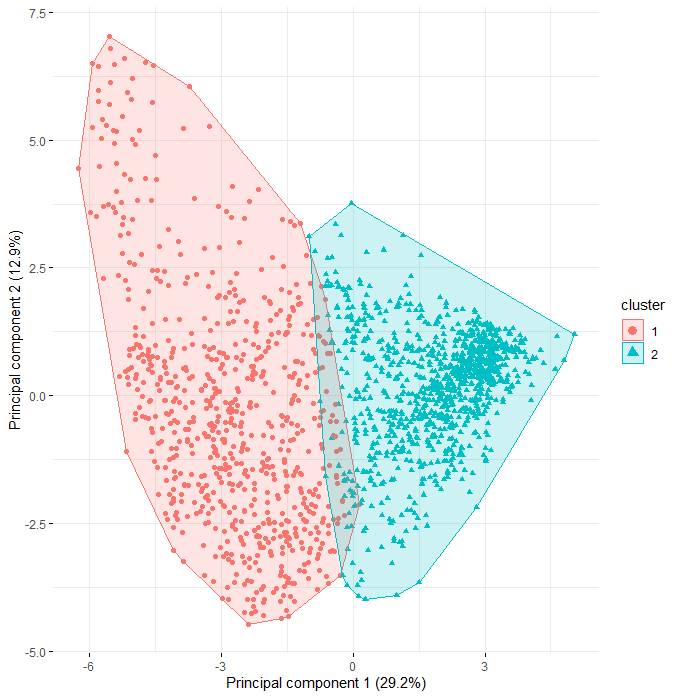


Supplemental Figure 2: Distribution of twins in the greater Helsinki area in 2012 by Clusters 1 (red) and 2 (green)


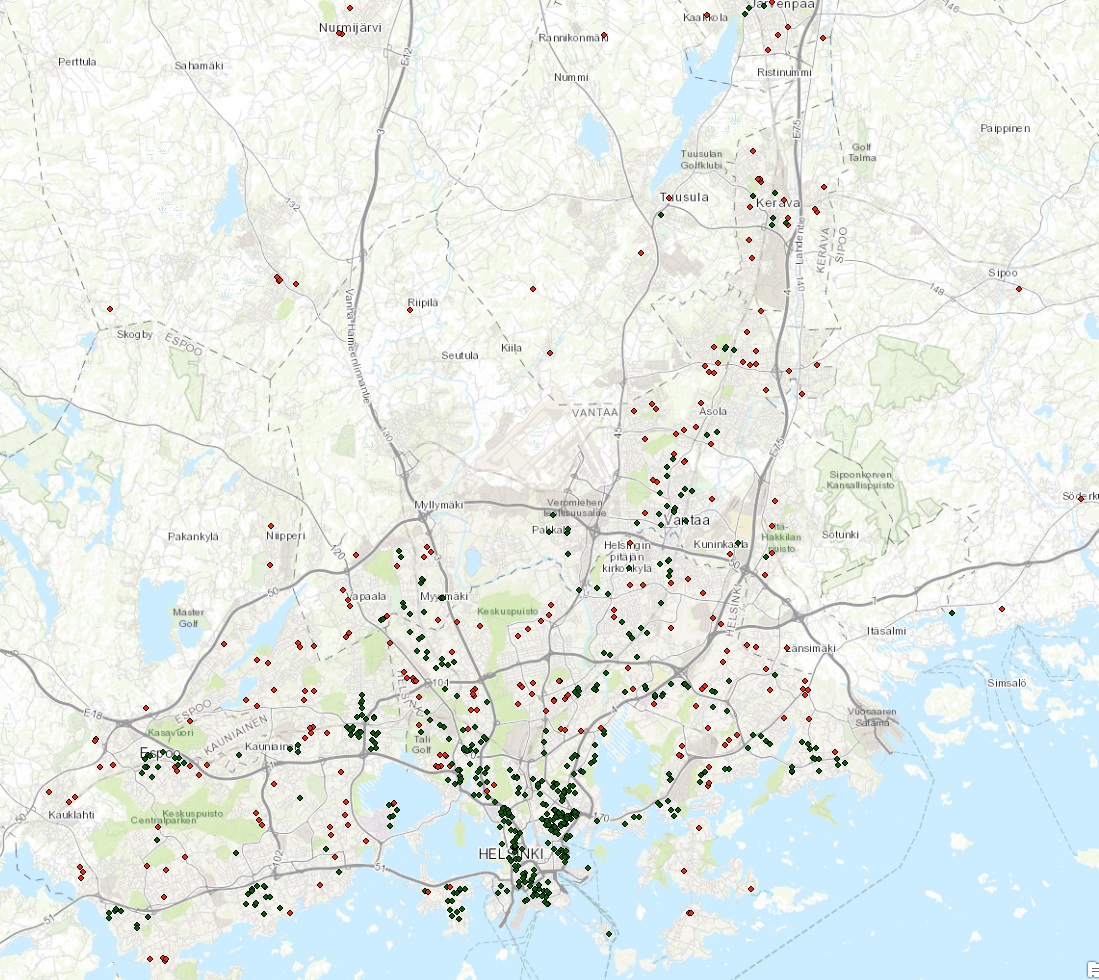


Supplemental Figure 3: Matrix of Pearson correlations between land use exposures based on the training subsets.


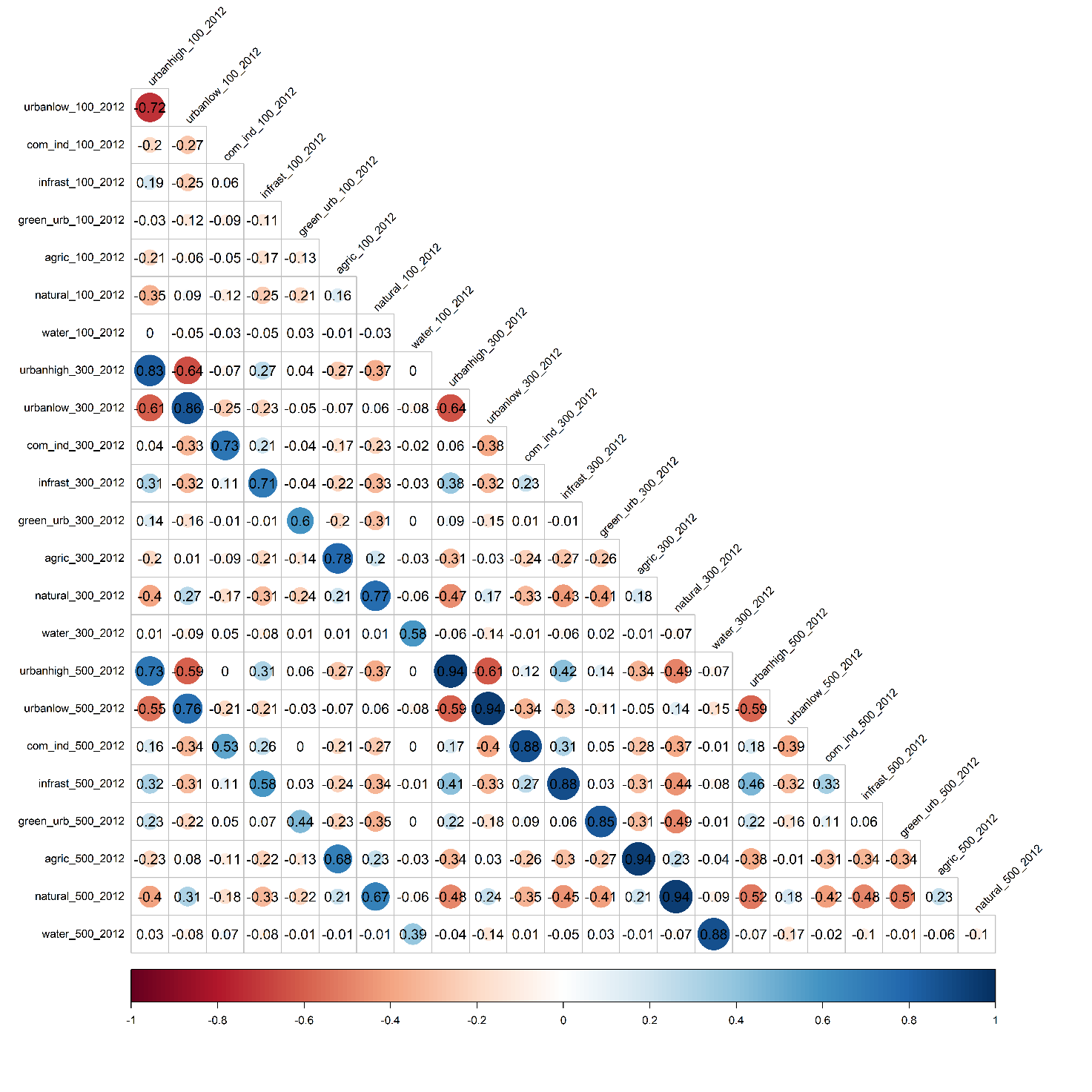


Note: The number indicates the correlation coefficient, the size of the circle indicates the size of the coefficient, and the blank (no color) indicates the lack of significance of the correlation coefficient.

Supplemental Figure 4: Distributions of the GBI score

Supplemental Figure 5: Shapley (SHAP) plots illustration of the top 5 most influential exposures in XGBoost models among overall participants after minimal (A) and further (B) adjustment.


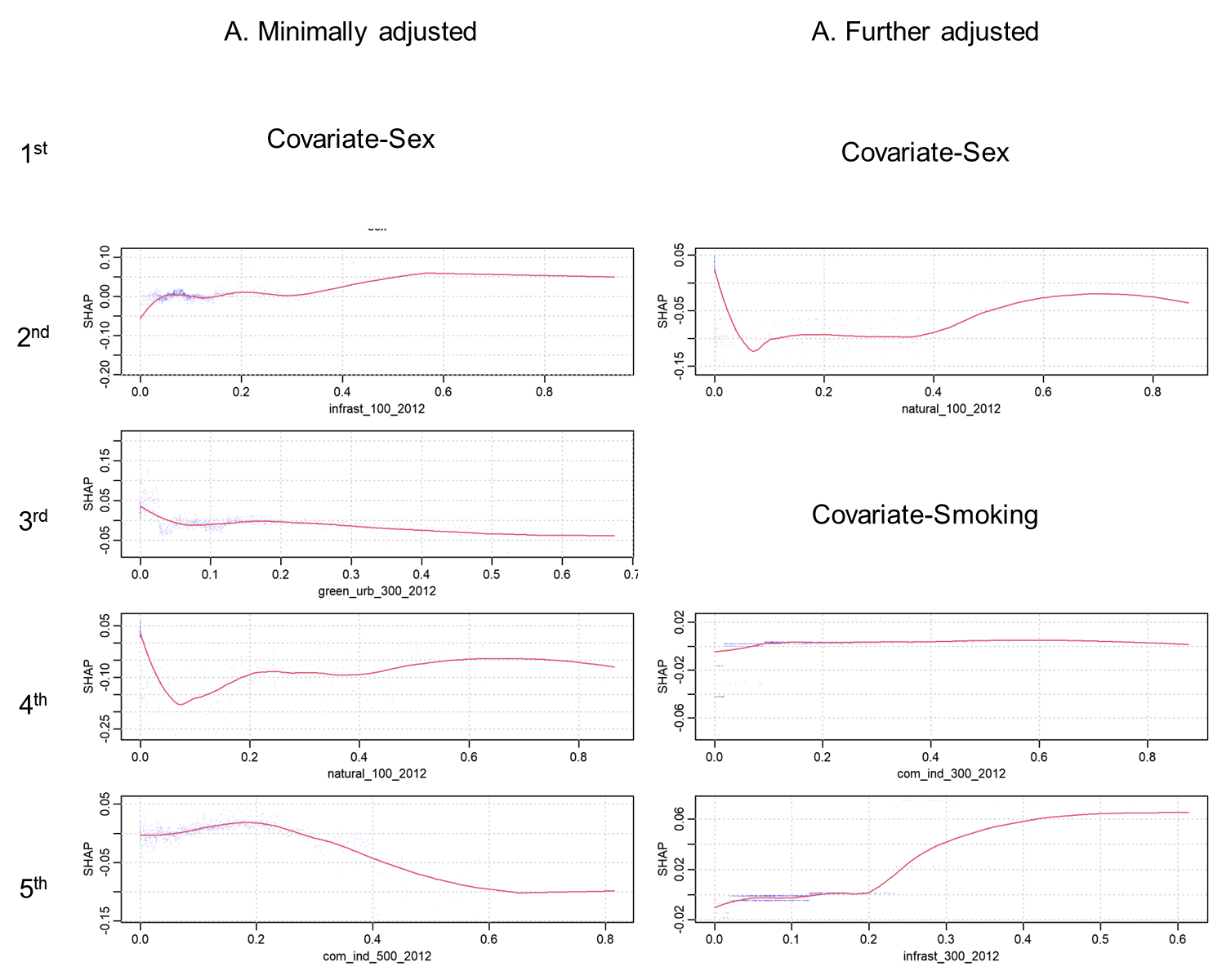


Note: Demographic covariates and social indicators were included in the models but suppressed in plots to highlight land use exposures.
